# Sleep and BMI: Do (Fitbit) bands aid?

**DOI:** 10.12688/f1000research.14774.2

**Published:** 2018-09-07

**Authors:** Laura McDonald, Faisal Mehmud, Sreeram V. Ramagopalan

**Affiliations:** 1 Centre for Observational Research and Data Sciences, Bristol-Myers Squibb, Uxbridge, UB8 1DH, UK; 2 Bristol-Myers Squibb, Uxbridge, UB8 1DH, UK

**Keywords:** sleep, BMI, fitbit, wearable

## Abstract

Recent studies have used mainstream consumer devices (Fitbit) to assess sleep objectively and test the well documented association between sleep and body mass index (BMI). In order to further investigate the applicability of Fitbit data for biomedical research across the globe, we analysed openly available Fitbit data from a largely Chinese population. We found that after adjusting for age, gender, race, and average number of steps taken per day, average hours of sleep per day was negatively associated with BMI (p=0.02), further demonstrating the significant potential for wearables in international scientific research.

## Introduction

The association between sleep and body mass index (BMI) is well known ^1^. Recently Xu and colleagues ^2^ showed that shorter sleep duration, as measured by a Fitbit wristband, was associated with a higher average BMI ^2^. These results importantly show the potential value of mainstream consumer devices for scientific research by providing objective sleep and physical activity data. A limitation of the Xu *et al.* study however, as noted by the authors ^2^, is the lack of diversity of ethnicity in their study population, with the majority of participants being of European descent. In order to assess the utility of wearables for global research we used data from a recently published study ^3^ to investigate the relationship between sleep and BMI in a largely Chinese population.

## Methods

Data was obtained from the study by Lim and colleagues ^3^. In brief, this study generated Fitbit Charge heart rate (HR) data from a cohort of volunteers tracked for a median duration of 4 days ^3^. The volunteers underwent comprehensive profiling including activity tracking (step count and sleep tracking) using the Fitbit Charge HR wearable sensor and BMI measurement at day of recruitment. Our criteria for ‘usable data’ in this study was based on the data availability from Lim *et al*. ^3^:

•   We started with the 223 participants Lim *et al*. ^3^ used in their analysis: “To ensure that results from various metrics are comparable, association analyses were conducted on a subset of subjects (223/233) with valid measurements for all metric types”

•   From 223 patients, we dropped 1 participant with missing diastolic blood pressure data, 1 participant with missing low-density lipoprotein data, and one participant with missing glucose data.

•   For the sleep pattern analysis we also looked into the standard deviation of daily sleep but six participants out of 220 had only one day of sleep data, therefore it was not possible to calculate a standard deviation of daily sleep duration for these patients. Two other participants did not have any sleep data in the sleep data file. The remaining 212 participants were included in our analyses.

To test the association between average hours of sleep and BMI multiple linear regression analyses were conducted using the ‘ statsmodels’ package in python.

One way to identify the minimum wear time necessary to get valid results from raw data is to run sensitivity analysis on data from participants who provided data for the full study duration, recalculate physical activity measures using data from fewer days from these patients, and calculate the intraclass correlation between physical activity status calculated from full data vs. partial data. When the correlation is above a pre-specified value, the appropriate number of days can be used as a cut-off point. Doherty *et al* ^4^. used this method to calculate a wear time criteria for their study of UK Biobank accelerometer study participants. Although we did not have access to more detailed raw data, we repeated a similar analysis on average daily sleep duration. Because only a small group of patients provided data for the full study duration, we made an assumption that at least six days of data can be considered ‘complete’. Then, using only sleep data from 54 participants who provided at least 6 days of data, we recalculated their average daily sleep duration using fewer days of data (average sleep duration based on only 1 day data, 2 days data, etc.). We thus obtained 6 average daily sleep durations for each of these 54 participants, calculated from different numbers of days. We then calculated the intraclass correlation (using the ICC function in the ‘psych’ package in R, see http://personality-project.org/r/html/ICC.html for details) of each of these average values with the values calculated from all available (at least six days in our sample) days.

## Results

A summary of participant clinical and demographic characteristics are shown in Table 1.

**Table 1.** Cohort clinical and demographic characteristics.

Characteristic	Value
Mean Age in Years (Standard Deviation, SD)	46.6 (12.1)
No. of Females (%)	123 (58%)
No. of Chinese (%)	195 (92%)
Mean Hours of Sleep (SD)	6.5 (1.1)
BMI (SD)	23.6 (4.1)
Mean daily steps (SD)	10826 (3865)

BMI: Body mass index

**Figure 1.**
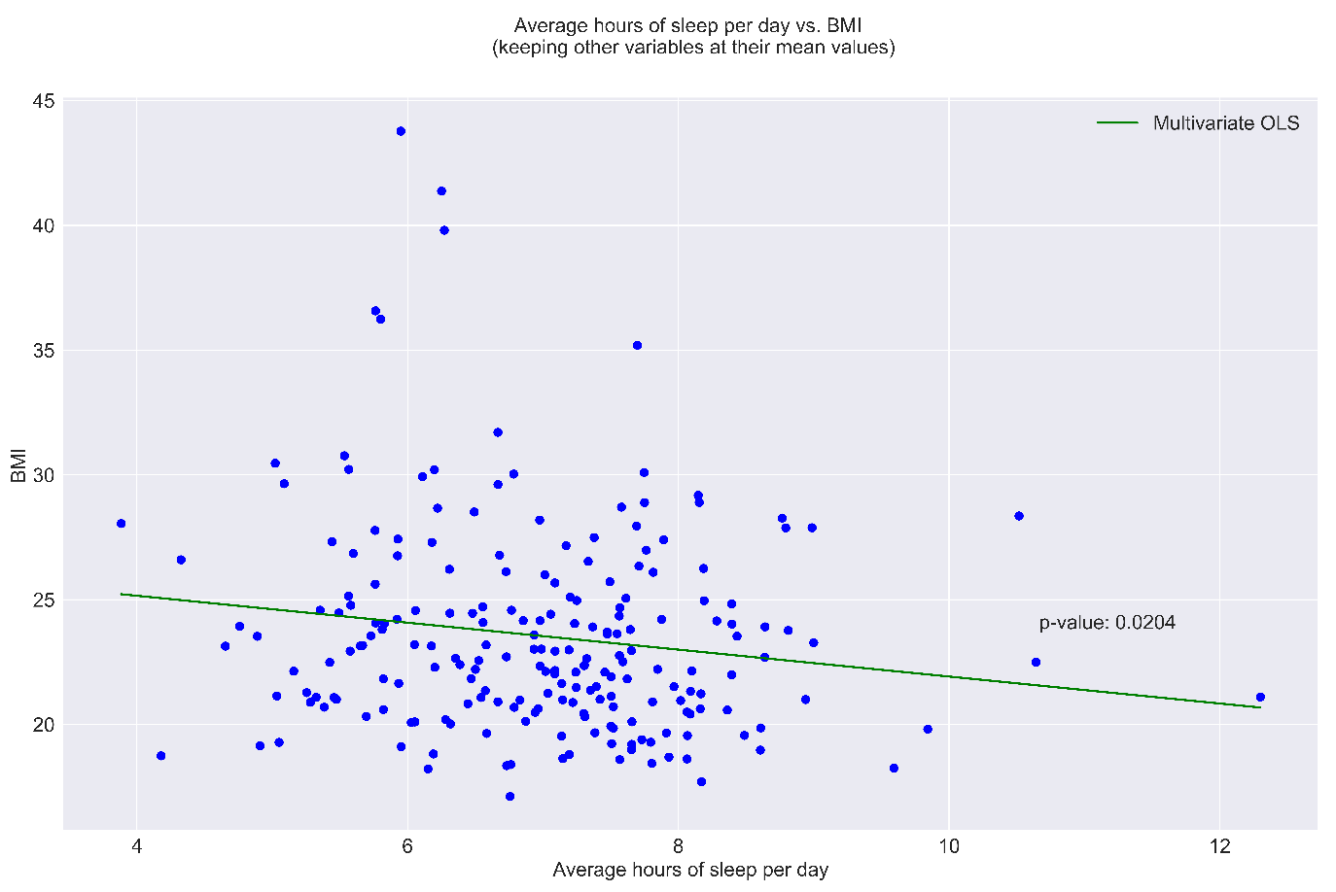
Relationship between body mass index (BMI) and average hours of sleep.

A linear regression analysis showed that after adjusting for age, gender, race, and average number of steps taken per day, average hours of sleep per day was negatively associated with BMI (p=0.02): an hour increase in sleep per day was associated with approximately a 0.5 point decrease in BMI ( Table 2, Figure 1).

**Table 2.** Multivariable linear regression analysis results for body mass index (BMI).

	Coefficient	P value
Intercept	29.21	<0.001
Age (per year increase)	0.0014	0.95
Sex (Male vs Female)	1.80	0.001
Race (Chinese vs other)	-3.35	0.001
Steps (per 1000 steps increase)	0.03	0.64
Average Sleep (per hour increase)	-0.54	0.02

Our sensitivity analysis showed that the ICC with 2 days of data was 80% with the ‘full’ data, rising to greater than 95% with 5 days of data. One day of data had an ICC of less than 60%.

## Conclusions

In summary, we found that the findings of Xu and colleagues are consistent in a population of different ancestry.

Compliance is one of the main challenges for gathering data with wearable technology. Patients might have difficulty charging or uploading data, might feel discomfort wearing the device, or simply forget to wear the device after removing it. But the high compliance rate in Lim *et al*. ^3^ suggest that wearables are a promising source of continuous and accurate data that can inform real life studies and clinical trials.

We tried to assess ‘how much’ data is needed for analyses such as the one we performed and our results suggest that at least 2 days of activity data is needed. Because we relied on the data made public by Lim *et al*. ^3^ we were not able to address other important elements of feasibility of collecting wearable data, such as battery life, security of data and privacy, and analytical methods to convert raw biosensor data to summary data (steps per minute, heart rate per minute, sleep duration, etc.) in this study.

To conclude, previous work ^2, 3^ and that described here demonstrates the significant potential for wearables in global biomedical research and further, as we used openly available data, this analysis shows the benefits of sharing observational data ^5^.

## Data availability

All data used in this study is available from the article by Lim *et al.* https://doi.org/10.1371/journal.pbio.2004285 ^3^
