# Supplementary material for: Sleep and BMI: Do (Fitbit) bands aid?
Source: F1000Res. 2018 Sep 7;7:511. Originally published 2018 Apr 27. [Version 2] doi: 10.12688/f1000research.14774.2 (PMC6144946; doi:10.12688/f1000research.14774.2)
Supplement: Peer review history

# Peer review: Referee response for version 2

**DOI:** 10.5256/f1000research.17734.r38088

**Published:** 2018-09-17

**Authors:** Maria R. Bonsignore

**Affiliations:** 1 Biomedical Department of Internal and Specialistic Medicine(DIBIMIS), University of Palermo, Palermo, Italy

Satisfactory response by authors to comments. Hope to see further studies on higher number of subjects to confirm the data.

I have read this submission. I believe that I have an appropriate level of expertise to confirm that it is of an acceptable scientific standard.

# Peer review: Referee response for version 2

**DOI:** 10.5256/f1000research.17734.r38087

**Published:** 2018-09-07

**Authors:** Eva Corpeleijn

**Affiliations:** 1 Department of Epidemiology, University Medical Center Groningen (UMCG), University of Groningen, Groningen, Netherlands

The amendments from version 1 are satisfactory.

I have read this submission. I believe that I have an appropriate level of expertise to confirm that it is of an acceptable scientific standard.

# Peer review: Referee response for version 1

**DOI:** 10.5256/f1000research.16075.r37363

**Published:** 2018-09-03

**Authors:** Maria R. Bonsignore

**Affiliations:** 1 Biomedical Department of Internal and Specialistic Medicine(DIBIMIS), University of Palermo, Palermo, Italy

I agree with the previous reviewer about methodological remarks. While wearable devices may help in collecting data and significantly contribute to generate hypotheses or confirm results, their reliability has not been rigorously tested.  An advantage of wearable devices is the possibility to collect large amount of data, which is not the case with this paper (n=212). Nevertheless, this work points to the possibility of increasingly available "big data", especially after appropriate validation studies.

I have read this submission. I believe that I have an appropriate level of expertise to confirm that it is of an acceptable scientific standard, however I have significant reservations, as outlined above.

# Peer review: Referee response for version 1

**DOI:** 10.5256/f1000research.16075.r35150

**Published:** 2018-08-16

**Authors:** Eva Corpeleijn

**Affiliations:** 1 Department of Epidemiology, University Medical Center Groningen (UMCG), University of Groningen, Groningen, Netherlands

The paper confirms a weak inverse association between sleep time and BMI using a mainstream consumer activity tracker (fitbit). The aim is to demonstrate the potential for wearables in scientific research.

 Because of this aim, it would be helpful to get additional information about feasibility aspects: what are the prerequisitions for usability in terms of data collection, how many of the participants had useful data based on which criteria, what strategies are needed for quality control to obtain meaningful associations?

 Definitions for 'useable data' should therefore be clarified.

 Sensitivity analyses can provide answers to what elements are important and what factors are secondary for a meaningful data use of consumer trackers.

I have read this submission. I believe that I have an appropriate level of expertise to confirm that it is of an acceptable scientific standard, however I have significant reservations, as outlined above.
